# The Influence of Mucin-Based Artificial Saliva on Properties of Polycaprolactone and Polylactide

**DOI:** 10.3390/polym11111880

**Published:** 2019-11-14

**Authors:** Dawid Łysik, Joanna Mystkowska, Grzegorz Markiewicz, Piotr Deptuła, Robert Bucki

**Affiliations:** 1 Institute of Biomedical Engineering, Bialystok University of Technology, Wiejska 45C, 15-351 Bialystok, Poland; g.markiewicz@doktoranci.pb.edu.pl; 2 Department of Microbiological and Nanobiomedical Engineering, Medical University of Bialystok, Mickiewicza 2C, 15-222 Bialystok, Poland; piotr.deptula@umb.edu.pl (P.D.); buckirobert@gmail.com (R.B.)

**Keywords:** degradation, saliva, mechanical properties, molecular weight, thermal properties, activation energy of thermal decomposition

## Abstract

Polycaprolactone (PCL) and polylactide (PLA) are the two most common biodegradable polymers with potential use in oral applications. Both polymers undergo mainly slow hydrolytic degradation in the human body. However, specific conditions of the oral cavity, like elevated temperature, low pH, and presence of saliva affect the rate of hydrolysis. The study examined the properties of solid samples of PCL and PLA subjected to degradation in phosphate buffered saline (PBS) and artificial saliva (AS) at temperatures of 37 or 42 °C, and pH values 2 or 7.4. A number of tests were performed, including measurement of the degree of swelling, weight loss, molecular weight, differential scanning calorimetry, and thermogravimetry of polymers, as well as hardness and tensile strength. Additionally, topography and stiffness of surfaces using atomic force microscopy are presented. It has been noticed that in the artificial saliva, the processes of polymer degradation occur slightly more slowly, and the effects of temperature and pH are less pronounced. We believe that a layer of porcine gastric mucin from artificial saliva that adsorbed on the surface of polymers may have a key role in the observed differences; this layer resembles protective mucin coating tissues in the oral cavity.

## 1. Introduction

Biomaterials are a wide group of materials used to evaluate, treat or replace tissues, organs or functions in the human body. In many areas of medicine, a paradigm shift from biostable materials to biodegradable materials has been observed in recent years. In dentistry, some of the permanent prosthetic devices used for temporary applications have been or will be changed to biodegradable products. The main reason for this situation is the biostability requirement of long-term implants and the need for revision surgery. The most important group of this type of applications are polymers [1,2,3,4]. Depending on the intended use, polymeric biomaterials may require a specific stability/degradation time. The time for which a polymer should retain the designed functions in the tissue environment, the so-called functional time, is the most important measure of its properties. Another, also important, parameter is the disappearance time, which determines the time for the total removal of material from the body. Between the functional time and the disappearance time, the biomaterial does not fulfill its functions. However, the polymer still releases degradation products, which, depending on the release rate and the physical and chemical properties, can trigger the body’s reactions [2]. The design of the polymer-based medical device requires determining the behavior of the material in the tissue environment. This is a difficult task because there are many factors affecting the degradation. In the bioactive environment of the human body, polymers can be decomposed during physicochemical and biological processes. This group contains hydrolysis, enzymatic or chemical (e.g., oxidation) reactions, and physical factors (including water absorption and swelling, fatigue by mechanical stresses, and wear). Usually, most of them act simultaneously and it is difficult to predict the behavior of the material *in vivo* [3,5]. Polymer degradation can be analyzed using complex mathematical models based on experimental data [6,7,8]. These models, however, are not able to predict the properties of the material during degradation, i.e., mechanical changes. If for various reasons, it is not possible to conduct *in vivo* experiments, then it is necessary to perform *in vitro* tests in conditions as close as possible to the properties of the implantation site.

The most promising group of biodegradable polymers are aliphatic polyesters, among which the most extensively investigated are polycaprolactone (PCL) and polylactide (PLA). PCL is a semi-crystalline polymer with a glass transition temperature from −60 to −55 °C and a low melting point of about 60 °C. PCL has low mechanical strength, but very high elongation at break (4700%), which makes it extremely flexible [9]. PCL is mainly used in tissue engineering for the regeneration of bone tissues [10], ligaments [11], cartilage [12], skin [13], nerves [14], and vessels [15]. Excellent processability of PCL allows scaffolding by electrospinning or by forming porous structures by treatment with a porogen. Due to slow degradation time, it is also used in long-term drug delivery systems, such as contraceptives [16].

Due to the fact that lactide is a chiral molecule and exist in two optically active forms—L-lactide and D-lactide—the polymerization of these monomers leads to the formation of semi-crystalline polymers: poly(L-lactide) (PLLA) and poly(D-lactide) (PDLA). The polymerization of racemic (D,L)-lactide and meso-lactide results in the formation of amorphous polymers poly(D,L-lactide) (PDLLA) and meso-polylactide [17]. Among them, PLLA and PDLLA are used for biomedical applications [18]. PLLA has good tensile strength and stiffness, its elastic modulus is 2.7 Gpa. The glass transition temperature is 60–65 °C and the melting point is 173–178 °C [19]. The time of complete resorption of PLLA is above 24 months, therefore it is most often used as a material for bone fixators [19]. It is also used in tissue engineering for the regeneration of bones [20,21], tendons [22], cartilage [23], nerves [24] and vessels [25,26]. Due to the slow degradation time, PLLA is not often used alone as a drug delivery system [27]. Sometimes, however, by modifying, mixing or copolymerization with other polymers (for example PGA), its degradation time is shortened [28,29,30,31]. PDLLA is an amorphous polymer due to the random positions of its two isomeric monomers in the chain, whereby the glass transition temperature is about 55–60 °C and the Young modulus is 1.9 Gpa [19]. The PDLLA full resorption time is shorter than PLLA resorption time and ranges from 12 to 16 months, therefore it is more often used as a drug delivery system [32] and in tissue engineering [33].

Due to their properties, such as biodegradability and ease of processing (common use in 3D printing [34]), PCL and PLA are used for oral cavity applications. According to the literature [35,36,37,38,39,40,41], they are used for scaffolds for trachea, teeth, and tooth–ligament interfaces, and as carriers for oral insulin delivery.

Both polymers in the human body undergo mainly slow hydrolytic degradation [5]. From a chemical point of view, PCL and PLA hydrolysis is a bimolecular nucleophilic substitution reaction that can be catalyzed by the presence of acids or bases [42]. The form of solid samples in which long chains are condensed means that the behavior of these polymers during degradation is more complicated than in other forms (microspheres, films). This is due to the fact that the reactivity in the crystal domains may be different than in amorphous ones [43,44]; the rate of degradation near chain ends may differ in comparison to other regions of the chain [45]. In addition, depending on the reaction rate and speed of molecule transport, surface erosion or bulk erosion may occur [46].

It is assumed that the kinetics of aliphatic polyesters’ degradation is the third order reaction and depends on the concentration of polymer bonds, water, and acid hydrolysis products [42]. According to this theory, there is a linear relationship between the logarithm of the polymer molecular weight and the degradation time, which was confirmed by the results of a series of experiments carried out by Pitt et al. [47,48] in which PCL and PLA degradation was examined *in vivo*. In the same studies, it was also observed that the weight loss of the samples was negligible until the molecular weight of PCL and PLA reached 5000 and 15,000 g/mol, respectively. Another feature of PCL and PLA hydrolysis is autocatalysis, whereby the degradation of solid samples is heterogeneous and occurs faster inside the sample than in the outer parts [49,50]. This feature is associated with the formation of the outer layer of slowly degrading polymer, whose macromolecules are trapped in the surface layer. Only oligomers with sufficiently low molecular weight are able to diffuse and dissolve. Inside the sample, however, the rapidly growing number of carboxyl groups accelerates the cleavage of ester bonds leading to auto-acceleration. This phenomenon was not observed in the pH buffered medium, which was thought to suggest that auto-acceleration was associated with low pH, although studies carried out in low-pH buffer solutions did not show enhancement of auto-acceleration [42]. Therefore, the effect of pH on degradation is still an open question, especially when we consider oral conditions in which pH variability is common. An important factor during degradation is also the temperature of the contact environment. In a few studies [51,52], it was confirmed according to the Arrhenius equation that with higher contact temperature, faster degradation process in the material is observed.

There are also studies on accelerated hydrolysis in the presence of some enzymes like pronase, proteinase K, or lipase [53,54,55,56,57]. However, specific conditions of the oral cavity, such as variability of pH and temperature, bacteria, fungi, and the presence of various types of chemical substances, affect the rate of degradation. On the other hand, biomaterials in direct contact with the oral environment coincide with a film with specific properties, mainly composed of macromolecules, such as mucins derived from saliva [58,59,60,61]. We believe that the conditions created by the saliva environment can affect the degradation of polymers in the oral cavity. In this work, we presented the influence of a mucin-based artificial saliva (AS) environment on degradation processes of PCL and PLA at temperatures of 37 and 42 °C, and pH values 2 and 7.4.

## 2. Materials and Methods

### 2.1. PCL and PLA Preparation for Degradation Studies

PCL (Sigma-Aldrich, Saint Louis, MO, USA) and PLA (3001D, D content 1.6%) (NatureWorks, Minnetonka, MN, USA) in the form of pellets were dried for 3 h. Then, on the Borche BS60 device (Borche, Rancho Cucamonga, CA, USA), samples of 30 mm × 5 mm × 4 mm dimensions were processed by injection molding. Next, obtained samples were conditioned at room temperature for 24 h (21 °C, 40% humidity). Two media were prepared for degradation studies: phosphate-buffered saline (PBS) (pH 7.4) and artificial saliva (AS) based on PBS, porcine gastric mucin (type III) (PGM), and xanthan gum (Sigma-Aldrich, Saint Louis, MO, USA) (pH 7.4), tested earlier [62,63]. Subsequently, the pH of half of the prepared medium was reduced to pH 2 by adding the appropriate volume of hydrochloric acid. The PCL and PLA samples were separately immersed in the medium in sealed, plastic containers and placed in two incubators, the internal temperatures of which were 37 ± 0.5 °C and 42 ± 0.5 °C. Designations adopted in the article are presented in Table 1. The whole cycle of tests lasted 8 weeks; every 5 days the medium was changed for a fresh one for each sample (n = 5). The polymers were tested for time intervals of 1, 2, 4, and 8 weeks.

### 2.2. Degree of Swelling, Weight Loss, and CLSM Observations

The degradation process of PCL and PLA was followed by determining weight loss and water absorption of the materials. Samples were washed with distilled water and gently wiped with paper. The degree of swelling was determined by comparing the wet weight (*w_w_*) with dry weight (*w_d_*) according to Equation (1):

(1) $$degree\;of\;swelling\left(\%\right)=\frac{w_{w}-w_{d}}{w_{d}}\times 100\%$$

The percentage of weight loss was determined after drying the samples (24 h, room temperature) by comparing dry weight (*w_d_*) with the initial weight (*w*_0_) according to Equation (2):

(2) $$weight\;loss\left(\%\right)=\frac{w_{0}-w_{d}}{w_{0}}\times 100\%$$

A balance (Mettler Toledo, Columbus, OH, USA) with a sensitivity of 0.01 mg was used to weigh the samples.

The surfaces of the samples were observed using a non-destructive real-time imaging technique: confocal laser scanning microscopy (CLSM) LEXT OLS 4000 (Olympus, Tokyo, Japan). For each test, five raw samples were tested. Samples were observed just after being rinsed three times in pure water to remove free molecules from polymer surface.

### 2.3. Molecular Weight

The molecular weight of polymers was estimated based on intrinsic viscosity measurements of polymer solutions in chloroform using iVisc Capillary Viscometer (Lauda-Brinkmann LP, Delran, NJ, USA) (Ubbelohde, capillary constant 0.003 mm^2^/s^2^). The Mark–Houwink equation, Equation (3), was used in the calculations:

(3) $$\left[\eta \right]=KM_{\eta }^{a}$$

where [η] is intrinsic viscosity, *M*_η_ is the estimated polymer molecular weight, K and *a* are constants of the equation which depend on polymer type, solvent, and temperature (for PCL-chloroform at 30 °C: K = 1.298∙10^−2^ g/cm^3^, *a* = 0.828 [64]; for PLA-chloroform at 25 °C: K = 6.06∙10^−2^ g/cm^3^, *a* = 0.64 [65]).

### 2.4. Differential Scanning Calorimetry (DSC) and Degree of Crystallinity

DSC studies were carried out using a DSC Discovery apparatus (TA Instruments, New Castle, DE, USA). The measurements were conducted in three cycles (heat–cool–heat), in a temperature range from −90 to 320 °C with a heating and cooling rate of 10 °C/min for PCL, and in a temperature range from −30 to 300 °C with a heating and cooling rate of 10°C/min for PLA. Results discussed in work were taken from second heating curves (first heating and cooling were performed to reduce the thermal history of tested samples). Three samples of each polymer were analyzed. Obtained DSC curves were used to analyze the glass transition temperature (*T*_g_), crystallization temperature (*T*_c_), cold crystallization enthalpy (Δ*H*_c_), melting temperature I, and fusion enthalpy (Δ*H*_m_). The degree of crystallinity (*X*_c_) for the samples was determined according to the following Equation (4):

(4) $$X_{c}=\frac{\Delta H_{m}-\Delta H_{c}}{\Delta H_{m}^{100}}\times 100\%$$

where Δ*H*_m_^100^ is fusion enthalpy of 100% crystalline PLA/PCL (93.7 J/g for PLA [66] and 135.3 J/g for PCL [67]).

### 2.5. Thermogravimetry (TG) and Activation Energy

TG tests were carried out using the Q500 thermogravimetric analyzer (TA Instruments, New Castle, DE, USA) in a temperature range from 30 to 500 °C and nitrogen atmosphere with heating rates (k) values of 5, 10, 20 °C/min. Special attention was paid to temperature at the start of thermal decomposition (*T*_DS_) (taken as 2% of the weight loss of the sample) and the end of thermal decomposition (*T*_DE_) (taken as 95% weight loss of the sample), and temperature at which the rate of weight loss was the highest (*T_max_*).

The Kissinger method was used to determine the activation energy of thermal decomposition of the tested materials. This method is based on the dependence of the temperature value *T_max_* (corresponding to the maximum of the DTG signal) to the heating rate *k* (Equation (5)):

(5) $$ln\left(\frac{k}{T_{max}^{2}}\right)=-\frac{E_{a}}{R}\times \frac{1}{T_{max}}+const.$$

As the heating rate increases, the temperature of the maximum intensity of the DTG signal also increases. The Kissinger method is based on presenting the obtained *T*_max_ values in the configuration ln(k/*T_max_*^2^)~1/*T_max_*. The directional coefficient of the obtained straight line corresponds to the value *E_a_*/*R* (where *E_a_* is activation energy, *R* is a gas constant equal to 8.31 J/mol∙K).

### 2.6. Hardness and Tensile Strength

Mechanical tests were carried out for wet samples, only washed with distilled water and wiped with paper. Hardness measurements of materials were performed using a Shore durometer (Type D) according to ASTM D2240. Tensile strength tests were carried out using a universal testing machine Zwick/Roell Z010 (Zwick Roell Group, Ulm, Germany) in accordance with ISO 527. Five samples of each polymer were analyzed for each test.

### 2.7. Atomic Force Microscopy Measurments

Topography and mechanical property measurements were recorded using an atomic force microscope (AFM) NanoWizard 4 BioScience AFM (JPK Instruments, Bruker, MA, USA) equipped with a liquid cell setup. Triangular-shaped cantilevers (AppNano NITRA-TALL-V-G) characterized by a spring constant of 0.37 N/m were used. Due to the lateral forces during contact mode scanning, a force curves-based imaging mode was used (JPK QI mode), with the resolution of 128 pixels per line, to show topography of the samples. QI maps also served as data for surface profiles and roughness examinations. Elastic modulus (i.e., the Young’s modulus) of the polymers was calculated based on force indentation curves from AFM, collected using the same cantilever. Elasticity maps size of 10 μm × 10 μm corresponding to a grid of 8 × 8 pixels and 25 μm × 25 μm areas corresponding to a grid of 16 × 16 pixels were carried out. Elasticity maps were collected from various samples areas. Young’s modulus was derived from the Hertz–Sneddon model applied to force-indentation curves.

## 3. Results and Discussion

At work, 8-week *in vitro* degradation studies of PCL and PLA samples, processed by injection molding were carried out. PBS or artificial saliva (AS, whose composition was based on mucin and xanthan gum) were used as the contacting environment. The main aim of this work was to verify whether the presence of macromolecules such as mucin and xanthan gum affects the process of polymer degradation. It was also examined whether the changes in pH and temperature of the environment, which reflect similar changes in the oral cavity and throat, will affect the degradation process. For this purpose, a number of physicochemical and mechanical tests were carried out, including evaluation of the material degree of swelling, weight and molecular weight change during degradation, hardness, tensile strength measurements, and DSC and TG thermal studies with the evaluation of the crystallinity of polymers and activation energy observed for polymers’ thermal decomposition. In addition, the layer resulting from the adsorption of macromolecules from the saliva was examined by atomic force microscopy to assess topography and mechanical properties.

### 3.1. Degree of Swelling, Weight Loss, and CLSM Observations

**Figure 1:**
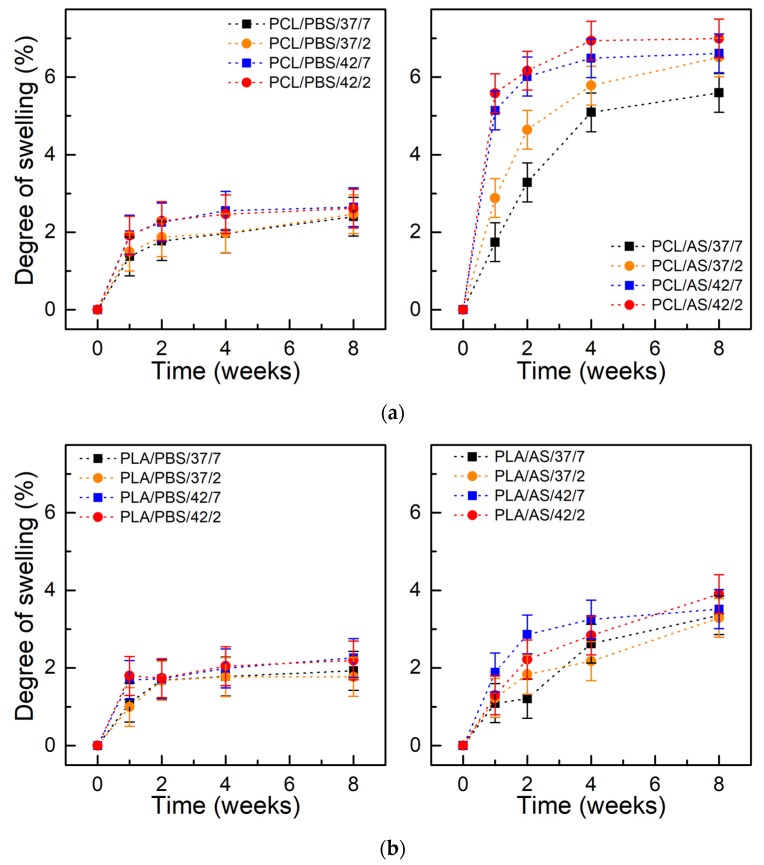
Swelling degree of materials during degradation: (**a**) polycaprolactone (PCL); (**b**) polylactide (PLA); (±SD, n = 5).

Figure 1 presents the degree of swelling of PCL and PLA as a function of incubation time. In case of artificial saliva, the absorption of PCL was 5–7% and was 3.5–4% for PLA after 8 weeks. For both materials, the degree of swelling was higher for samples incubated in AS. Higher swelling degree probably mainly results from the presence of the adsorbed layer of macromolecules on the surface of samples, which has not been washed away, than from the penetration of mucin and xanthan gum molecules into the bulk of the material. In both materials, the degree of swelling was higher at 42 °C than at 37 °C, which results from faster diffusion of water into the material at a higher temperature. In addition, it was observed that the highest increase of absorption occurred at the beginning of the degradation period (2–4 weeks), in subsequent time intervals a plateau in level was noticed.

**Figure 2:**
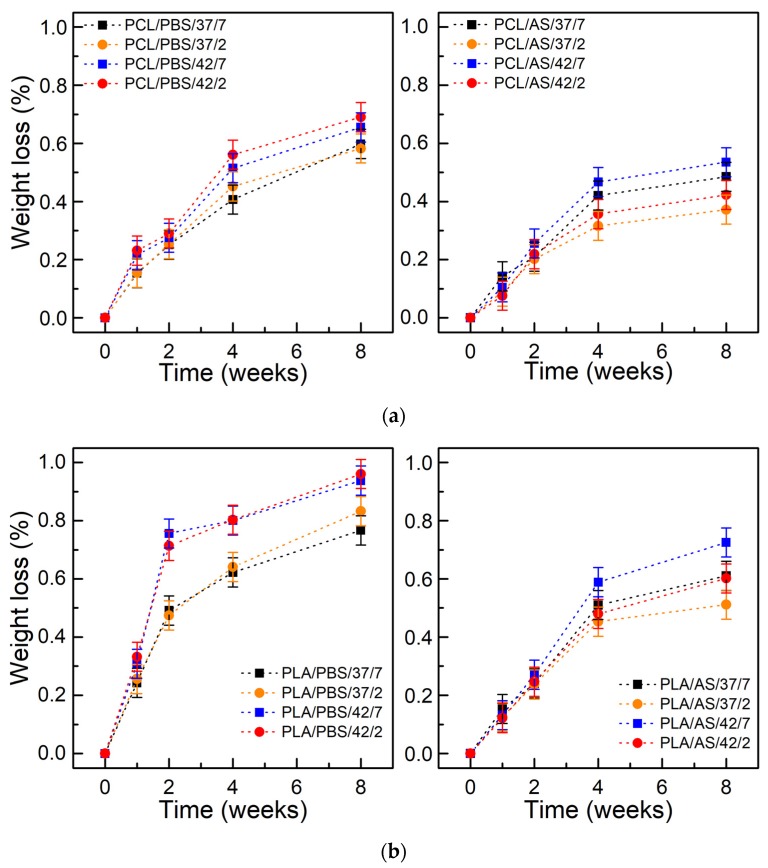
Weight loss of materials during degradation: (**a**) PCL; (**b**) PLA; (±SD, n = 5).

Figure 2 shows the weight loss as a function of degradation time. After 8 weeks the weight loss of PCL incubated in PBS reached 0.5–0.7% and reached 0.3–0.5% for PCL incubated in the AS. In case of PLA, weight loss after 8 weeks reached 0.75–0.95% in PBS and reached 0.45–0.7% in AS. It was observed that there is a correlation between swelling degree and weight loss (in frame of each polymer and type of environment). Hydrolysis is the major mechanism of aliphatic polyester degradation; PCL and PLA belong to this group. The rate of its hydrolytic degradation is determined by the water concentration, and thus the degree of swelling. The polyesters can absorb up to 1% water, however, due to hydrolysis, more hydrophilic chain ends are formed, and polymers may absorb increasing amounts of water. The kinetics of hydrolytic degradation, therefore, depend on the diffusion coefficient of water to the polymer, which for polyesters, regardless of the state, is about 10^−8^ cm^2^/s (at 37 °C), which can be compared to penetrating 1 mm of the sample in a few days [5]. The coefficient of water diffusion (and the diffusion coefficient of chain fragments within the polymer, and the solubility of degradation products) through the polymer increases with temperature, which agrees with the observations obtained in this study. At 42 °C, the weight loss was higher than at 37 °C.

The weight loss of both materials was lower in the artificial saliva when compared to PBS. We believe that the mucin layer formed on polymers’ surface, like in the oral cavity (or the respiratory/gastrointestinal tract) [68], covers the surfaces of materials and creates a protective layer [61,62]. It can act as a barrier and finally slow down the degradation of polymers.

For materials incubated in PBS at different pH values, 2 and 7.4, small differences were observed either in the degree of swelling or in weight loss. The influence of pH in aqueous solutions on the rate of degradation has been studied in several works [5,69,70,71,72]. Studies of PLA dissolved in tetrahydrofuran with a pH in the range of 0–14 showed the slowest degradation at pH 4 [5]. Other studies [69] on the degradation of PLA polymer chains attached to the surface (brush structure) in the pH range 3–8 showed the slowest degradation at pH 3. PLA degradation is the slowest in solutions with pH 4, because pKa of lactic acid is 3.84. In solutions with pH > 4, lactic acid is in dissociated form, which favors faster hydrolysis. In solutions with pH < 4, lactic acids at the chain ends prefer the associated acid form, which also accelerates the hydrolysis [5]. However, in solid polymers, the effect of pH on the rate of degradation is often negligible [5,71]. This is due to the low solubility of H^+^ and OH^−^ ions in polymers so that they cannot effectively affect degradation.

For both materials incubated in artificial saliva at different pH levels, the differences were visible, and the weight loss of polymers contacted with solutions with pH 2 was lower. This may be due to the specific properties of mucin, which under the influence of low pH, can change their conformation, making the created layer a stronger barrier.

**Figure 3:**
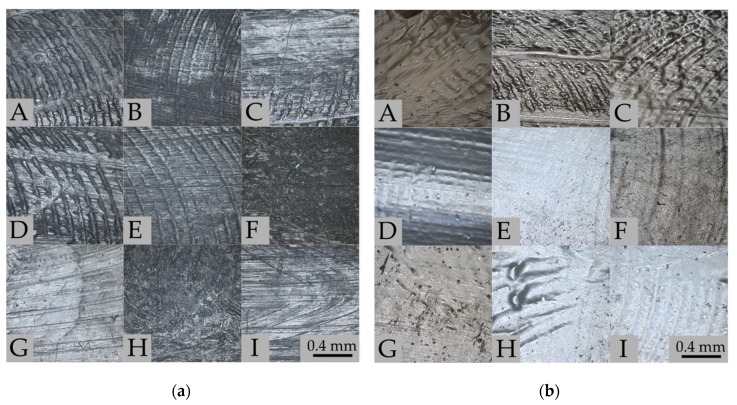
Surfaces of tested polymers before and after 8 weeks degradation imaged using CLSM: (**a**) PCL—before degradation (A); PBS/37/7 (B); PBS/37/2 (C); PBS/42/7 (D); PBS/42/2 (E); AS/37/7 (F); AS/37/2 (G); AS/42/7 (H); AS/42/2 (I). (**b**) PLA—before degradation (A); PBS/37/7 (B); PBS/37/2 (C); PBS/42/7 (D); PBS/42/2 (E); AS/37/7 (F); AS/37/2 (G); AS/42/7 (H); AS/42/2 (I).

Figure 3a shows the surfaces of PCL samples before (panel A) and after 8 weeks of degradation in PBS (panels B–E) and artificial saliva (panels F–I). CLSM observations were performed on identical parts of the samples. Before degradation tests (Figure 3a, panel A) a characteristic texture is visible for elements prepared by the injection molding method. After 8 weeks of degradation, the surface texture is less visible, which may indicate that surface erosion has occurred. In samples F and H, which present samples after degradation in artificial saliva at pH = 7.4, there are probably residues on the macromolecular layer adsorbed on the surface. Figure 3b presents the surface of the PLA samples before (panel A) and after 8 weeks of degradation in PBS (panels B–E) and artificial saliva (panels F–I) solutions. Significant differences are visible between the surfaces of the tested polymer samples. In samples B and C, the surface morphology is more complex than in sample A, the characteristic striated texture has been enhanced. The surfaces for samples E, F, H, and I after degradation at 42 °C have changed to “milky” color and the surface is smoother due to erosion; these results correlate with weight loss. However, in samples F and G there are visible residues from the adsorbed layer of macromolecules (which correlates with results of swelling).

### 3.2. Molecular Weight

**Figure 4:**
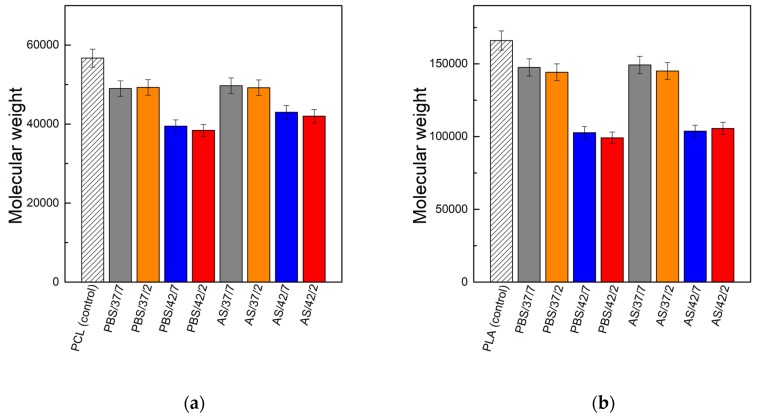
Molecular weight of polymers before (control) and after 8-weeks of degradation: (**a**) PCL; (**b**) PLA.

Figure 4 presents the results of molecular weight measurements of polymers PCL and PLA (estimated on the basis of intrinsic viscosity measurements) after 8 weeks of degradation in the PBS and artificial saliva in relation to the molecular weight of non-degraded polymer samples. The molecular weight of PCL samples kept in PBS solution at 37 °C decreased after 8 weeks by about 13%; at 42 °C the decrease was much higher—about 30–32%. The artificial saliva slightly affected the decrease of molecular weight in relation to the PBS. At 37 °C about 12–13% of molecular weight loss was observed; at 42 °C about 24–26% of molecular weight loss was observed. Similar trends were observed for PLA. In the case of PBS environment at 37 °C the decrease in molecular weight was about 11–13%, and at 42 °C, the decrease was about 38–40%. For the artificial saliva at 37 °C, molecular weight decrease was 10–12%; at 42 °C it was 36–37%. For both polymers and both contact solutions, no significant differences between low and neutral pH were observed (these differences were a maximum of 2% of molecular weight loss). The observed trends correlate with the results of weight loss, where a lower decrease of mass in the environment of artificial saliva was also observed. In case of this medium, an increase of temperature had a much greater impact on the degradation process of polymers in comparison to the influence of the pH of the contact environment.

### 3.3. Thermal Studies

Thermal tests using DSC and TG are very helpful in assessing changes in the material, especially in the initial stage of degradation, which are difficult to investigate by other methods. There are few works in which the glass transition temperature, melting temperature, or the degree of crystallinity calculated on the basis of the enthalpy of physical transformations were used to monitor the degradation process. Reich [73] observed that the glass transition temperature and the melting temperature of the PLA microparticles decreases due to degradation process. In the work of Paul et al. [74], it has been shown that the decrease in molecular weight during hydrolysis is accompanied by decreases in the glass transition and crystallization temperatures, as well as an increase in the degree of crystallinity.

The thermal and crystalline properties of the PCL and PLA before and after the degradation period obtained from the DSC and TG curves are shown in Table 2. For both materials, changes in thermal properties were observed. For PCL, there was a significant reduction in glass transition temperature from the initial value of −65.2 °C to about −80 °C for samples kept in the AS. In the case of PLA, a significant decrease in the glass transition temperature was observed for samples PLA/PBS/42/7 and PCL/AS/42/2 (from initial value 48.5 °C to below 42 °C). The decrease in *T*_g_ is related to both the reduction of molecular weight and the plasticizing effect by acid oligomers formed during degradation.

In both materials, degradation decreased the value of crystallization temperature. In case of PCL, this was from the initial value of 37.5 °C to below 36 °C for samples PCL/PBS/42/7 and PCL/PBS/42/2, and for PLA with an initial value of 107.7 °C, this decreased to even 95 °C for PLA/AS/42/2. The decrease in the crystallization temperature can be interpreted as a decrease in molecular weight since shorter polymer chains tend to crystallize at lower temperature [74].

The melting temperature was also generally reduced after degradation. The highest changes were observed for PLA samples aged in PBS at 42 °C (from initial 146.7 °C to about 135 °C). For PCL, changes in the melting temperature were lower than for PLA. Thus, it can be concluded that the observed changes were irrelevant (melting point for all samples was in the range 53–54 °C).

The evolution of the global crystallinity *X*_c_ of the PLA and PCL during hydrolysis can be deduced through the evolution of both Δ*H*_c_ and Δ*H*_m_. PLA has a generally low crystallinity which indicates a quasi-amorphous structure. As a result of degradation performed in this study, its crystallinity increases, but these are rather small changes (from the initial 2.6% to a maximum of 4.6%). PCL is a semi-crystalline polymer and due to degradation its crystallinity also increases, from the initial 59.3% to a maximum of 65.5%. Due to the fact that the range of changes in the weight of samples during degradation was low (up to 1%), the increase in crystallinity cannot be attributed to the change of the ratio of the crystalline phase to amorphous phase (the amorphous phase is generally decomposed faster than the crystalline one). This increase has to be attributed to an effective crystallization occurring during degradation. A possible mechanism of such a process may be an increase of the polymer chains’ mobility due to the action of water, PBS medium, saliva macromolecules, and resulting degradation products (by the decrease of the chains’ molecular weight and plasticization), which could result in structure reorganization and crystallization [74].

Hydrolytic degradation affects the thermal stability of polymers, often determined by the temperature of the start (*T*_DS_) and end (*T*_DE_) of thermal decomposition. In the case of untreated PCL, the temperatures were 360.6 °C and 450.9 °C, respectively. After 8 weeks of degradation, PCL had a higher *T*_DS_ (361.4–367.1 °C) and lower *T*_DE_ (426.8–450.6 °C) in all tested conditions. However, there were no differences between individual environments. In the case of PLA, for an unaged sample, *T*_DS_ was 324.2 °C and *T*_DE_ was 388.7 °C. It may be concluded that after degradation both *T*_DS_ (308.9–319.8 °C) and *T*_DE_ (378.5–385.9 °C) were decreased. Samples incubated in artificial saliva were characterized by a lower *T*_DS_. Decrease of both temperatures is a result of molecular mass decrease. When considering the type of environment, the surface mucin-based saliva layer may decrease the rate of degradation.

The kinetics of the thermal decomposition process depend on the structure of the polymer and molecular weight. Therefore, structural changes due to hydrolysis may be indirectly observed by determining the activation energy of thermal decomposition. The activation energies of polymers measured before and after incubation in contact solutions were 221.2 kJ/mol (before) and 160.3–218.8 kJ/mol (after incubation in PBS and AS) for PCL; and 190.2 kJ/mol (before) and 144.0–177.2 kJ/mol (after incubation in PBS and AS) for PLA. After degradation in various tested environments, the activation energy generally decreased, which means that less energy is required to initiate thermal decomposition of tested materials. In the case of degradation performed in PBS, the greatest decreases of activation energy were noted for the environment at pH 2 (samples PCL/PBS/42/2, PLA/PBS/37/2, and PLA/PBS/42/2) in comparison to pH 7.4. The increase of contact environment temperature reduced the activation energy of the thermal decomposition process for both polymers.

### 3.4. Mechanical Tests

Water absorption, weight loss, and molecular weight loss as a function of time, either *in vivo* or *in vitro* studies in simulated body fluids, are useful parameters for assessing the behavior of biodegradable materials. Implants in the body environment often require the preservation of their mechanical properties, such as hardness, stiffness, tensile, compression, or bending strength. While the increase in water sorption, the weight loss, and the decrease of polymer molecular weight are obvious, and the conducted research gives information about the rate of these processes, changes in mechanical properties are more difficult to predict. In addition, the loss of weight and molecular weight does not always correlate with the decrease in mechanical properties—especially in the initial stages of the degradation process.

**Figure 5:**
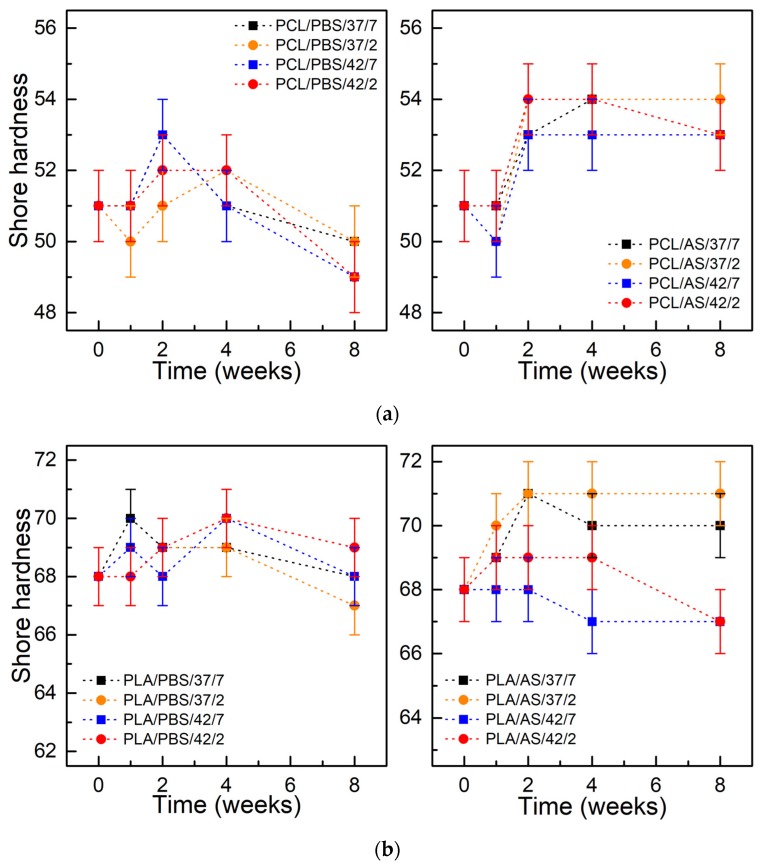
Shore hardness of materials during degradation: (**a**) PCL; (**b**) PLA; (±SD, n = 5).

Figure 5 shows the Shore hardness of PCL and PLA as a function of degradation time. For both materials incubated in PBS and AS solutions, a slight increase of hardness during degradation up to 4 weeks was observed, followed by a slight drop to the initial or lower values at the end of the considered period. The higher hardness was observed in the case of materials kept in the environment of artificial saliva, which correlates with lower weight loss.

**Figure 6:**
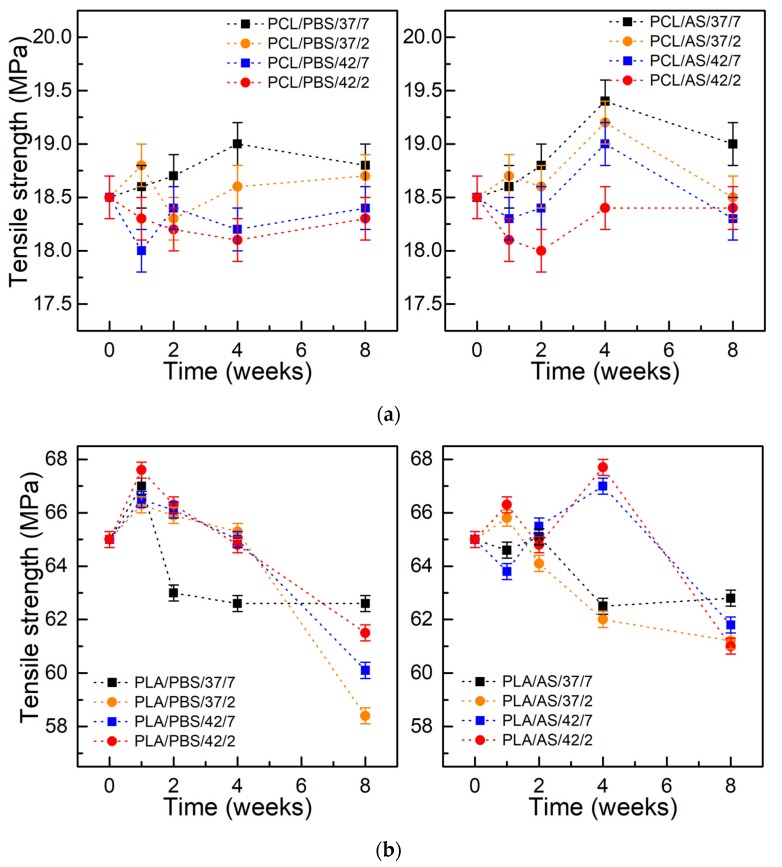
Tensile strength of materials during degradation: (**a**) PCL; (**b**) PLA; (±SD, n = 5).

Figure 6 presents the tensile strength of PCL and PLA samples as a function of degradation time. Similarly to the case of hardness tests, a slight increase in tensile strength was observed over the first 4 weeks of testing, then a gentle decrease was observed. In case of PCL immersed in PBS, the value of the tensile strength after 8 weeks did not change and, depending on the pH and temperature of the environment, it was within 18.25–18.75 MPa (initial value was 18.5 MPa). For PCL kept in AS, the tensile strength after 8 weeks was 19 MPa for a medium with pH 7.4 and temperature 37 °C, and about 18.5 MPa for the remaining conditions. For PLA immersed in PBS, a slight increase of tensile strength was observed after the first week of degradation from an initial value of 65 MPa to about 68 MPa for the PLA/PBS/42/2 sample. In the next weeks, a continuous decrease in the value of up to 62 MPA for the PLA/PBS/37/7 sample and around 58 MPa for the PLA/PBS/37/2 sample were observed. For the PLA kept in the AS environment, a decrease in the tensile strength value up to 61–62 MPa was also observed, however, preceded by an increase in the 4th week for samples kept at 42 °C.

During the degradation process of both PCL and PLA, an increase in the mechanical strength was initially perceived, which is caused by the absorption of water. An increase of hydrogen bond formation restricts the movement of chains past each other, and a consequential increase of the tensile strength during first 4 weeks of testing was observed. After strengthening the material, further absorption of water leads to polymer chains shortening. As a result, a decrease of mechanical properties (tensile strength, hardness) occurred. A similar character of mechanical changes was noticed in a few other works. In [75], after 30 days of hydrolytic degradation, a 6% increase in tensile strength and a 3% increase in Young’s modulus were observed, after which a significant decrease in mechanical strength was noticed. In [76], the *in vivo* degradation of PLA implants for bone fixation was investigated. Until the 12th week, a slight increase in the mechanical strength of the examined implants was observed; 36 weeks after implantation, the mechanical strength was reduced by 23%.

### 3.5. AFM Measurments

**Figure 7:**
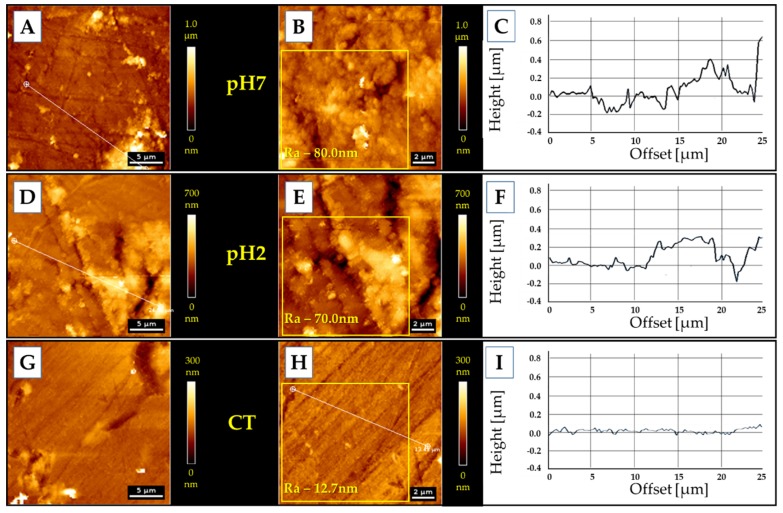
Topography of polymer surfaces: (**A**–**C**) the samples kept in pH 7 artificial saliva; (**D**–**F**) samples kept in pH 2 artificial saliva; (**G**–**I**) the control sample.

**Figure 8:**
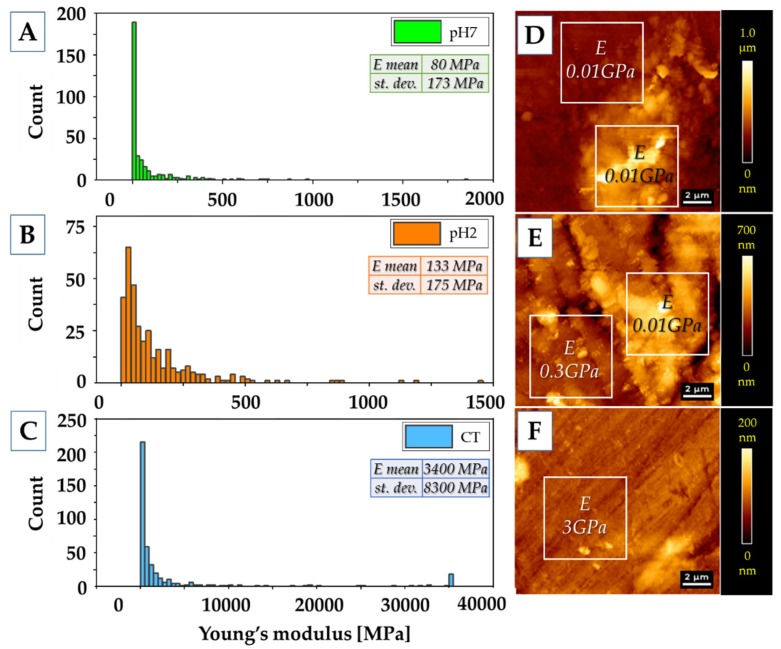
Mechanical properties of the polymer surfaces. (**A**–**C**) Young’s modulus values distributions. (**A**) pH 7 sample; (**B**) pH 2 sample; (**C**) control sample. Inside the panels, mean values of Young’s modulus (E) and standard deviations (st. dev.) are presented. (**D**–**F**) Topography of the samples’ surfaces with values of Young’s modulus from two areas—flat surface and high additional layer. (**D**) pH 7 sample; (**E**) pH 2 sample; (**F**) control sample.

To assess the properties of the thin layer of mucin adsorbed on the surface of samples during incubation in artificial saliva (pH 7.4 and pH 2) for 7 days, AFM measurements of topography (Figure 7) and mechanical properties (Figure 8) were carried out.

An adsorbed layer (Figure 7A,B,D,E) is visible on the surface of samples incubated in an artificial saliva, which changes the surface topography to a more rough one—from Ra = 12.7 nm (control sample) to Ra = 80 nm and Ra = 70 nm, for pH 7 and pH 2, respectively. The height of the layer formed at pH 7 is about 400 nm; at pH 2 the height is about 300 nm.

The adsorbed layer of mucin is definitely less stiff (80–133 MPa) than the polymer surface, whose Young’s modulus was estimated at 3 GPa. However, it is so stiff and compact that it can be a barrier similar to that created by mucus on the tissues/biomaterials. Moreover, the layer formed on polymers incubated at lower pH is stiffer than that created in a neutral pH environment. Similar trends were observed ex vivo via AFM on fresh mucus-covered tissues [77]. The mucus tested at lower pH was not only stiffer, but also less adhesive. Using dynamic light scattering studies [78] it was demonstrated that pH lowering results in conformational porcine gastric mucin transition from the isotropic random coil at pH 7 to the anisotropic extended random coil at pH 2. Lowering pH results in a significant increase of viscosity and viscoelasticity of solutions as well as concentrated PGM [79], [80]. This transition is driven by a decrease in electrostatic repulsion between anionic fragments of mucin molecules, which allows interaction and aggregation of their hydrophobic residues [77].

## 4. Summary and Conclusions

The study investigated the PCL and PLA—two most common biodegradable polymers with potential use in oral applications. Degradation was carried out in two contact solutions—PBS and artificial saliva based on porcine gastric mucin and xanthan gum. The results of sample weight loss and molecular weight loss indicate a greater degree of polymer degradation in the PBS medium than in the artificial saliva environment. It may be concluded that the layer of mucin-based saliva acts as a barrier, and finally, a decrease in the degradation of polymers is observed. In addition, a significant effect of elevated temperature on the rate of degradation was observed, particularly evident in the results of molecular weight studies, in which a greater decrease was observed in case of higher temperature. However, lowering the pH of the environment had a lesser impact on the degradation process. An increase of temperature had a much greater impact on the degradation process of polymers in comparison to the influence of the pH of the contact environment. The increase of contact solution temperature reduced the activation energy of the thermal decomposition process for the tested polymers. The observed changes in physicochemical and thermal tests reflect the results of tests of mechanical properties. An increase of mechanical properties during the first 4 weeks of testing was observed. Further water absorption leads to the decrease of tested mechanical parameters.

The macromolecule layer formed on the surface of polymers can affect the rate of degradation. On the other hand, in applications such as drug carriers, the resulting macromolecule layer may impede the release of the substance from polymer.

## Figures and Tables

**Table 1:** Designations used in the work.

Designation	Material	Medium	Temperature (°C)	Ph
PCL/PBS/37/7	PCL	PBS	37	7.4
PCL/PBS/37/2	PCL	PBS	37	2
PCL/PBS/42/7	PCL	PBS	42	7.4
PCL/PBS/42/2	PCL	PBS	42	2
PCL/AS/37/7	PCL	Artificial saliva	37	7.4
PCL/AS/37/2	PCL	Artificial saliva	37	2
PCL/AS/42/7	PCL	Artificial saliva	42	7.4
PCL/AS/42/2	PCL	Artificial saliva	42	2
PLA/PBS/37/7	PLA	PBS	37	7.4
PLA/PBS/37/2	PLA	PBS	37	2
PLA/PBS/42/7	PLA	PBS	42	7.4
PLA/PBS/42/2	PLA	PBS	42	2
PLA/AS/37/7	PLA	Artificial saliva	37	7.4
PLA/AS/37/2	PLA	Artificial saliva	37	2
PLA/AS/42/7	PLA	Artificial saliva	42	7.4
PLA/AS/42/2	PLA	Artificial saliva	42	2

**Table 2:** Glass transition temperature (*T*_g_), crystallization temperature (*T*_c_), melting temperature (*T*_m_), and crystallinity (*X*_c_) obtained from DSC curves, along with starting thermal decomposition temperature (TDS), end of thermal decomposition temperature (*T*_DE_), and activation energy of thermal decomposition (*E*_a_) obtained from TG of PCL and PLA after 8 weeks of degradation.

Sample	*T*_g_ (°C)	*T*_c_ (°C)	*T*_m_ (°C)	*X*_c_ (%)	*T*_DS_ (°C)	*T*_DE_ (°C)	*E*_a_ (kJ/mol)
PCL	−65.2	37.5	54	59.3	360.6	450.9	221.2
PCL/PBS/37/7	−70.6	36.4	54.8	59.6	361.4	450.6	218.8
PCL/PBS/37/2	−65.3	36.1	53.5	59.9	365.2	436.7	174.4
PCL/PBS/42/7	−65.3	35.5	53.2	65.5	365.6	433.3	183.8
PCL/PBS/42/2	−69.95	35.1	53.5	63.6	367.1	433.2	160.3
PCL/AS/37/7	−78.3	36.7	53.8	57.8	364.3	426.8	162.7
PCL/AS/37/2	−79.7	36.8	53.8	59.0	366.4	434.9	181.3
PCL/AS/42/7	−79.4	36.4	53.4	60.0	365.3	434.1	177.6
PCL/AS/42/2	−82.2	36.7	53.1	61.5	366.4	434.4	177.9
PLA	48.5	107.7	146.7	2.6	324.2	388.7	190.2
PLA/PBS/37/7	46.8	96.9	143.7	3.2	319.8	385.9	163.0
PLA/PBS/37/2	47.1	97.6	145.6	3.1	319.7	385.2	140.4
PLA/PBS/42/7	41.1	103.5	134.5	2.8	316.9	384.7	154.2
PLA/PBS/42/2	41.6	100	135.5	2.6	316.6	384.4	144.0
PLA/AS/37/7	47.3	96.6	144.2	3.1	308.9	378.5	158.2
PLA/AS/37/2	46.5	99.2	141.7	3.5	318.0	385.1	170.1
PLA/AS/42/7	45.7	99.3	140.2	4.2	314.1	382.1	157.1
PLA/AS/42/2	47.5	95	145.3	4.6	310.7	383.0	177.2
